# A Prospective Study of High Dose-Rate Brachytherapy or Stereotactic Body Radiotherapy of Intra-Prostatic Recurrence: Toxicity and Long Term Clinical Outcome

**DOI:** 10.3389/fonc.2022.861127

**Published:** 2022-04-05

**Authors:** Una Ryg, Therese Seierstad, Line Brennhaug Nilsen, Taran Paulsen Hellebust, Linda Holth Djupvik, Hilde Gustafson, Jørgen Hydal, Amar U. Kishan, Knut Håkon Hole, Wolfgang Lilleby

**Affiliations:** ^1^ Division of Radiology and Nuclear Medicine, Oslo University Hospital, Oslo, Norway; ^2^ Department of Medical Physics, Oslo University Hospital, Oslo, Norway; ^3^ Department of Physics, University of Oslo, Oslo, Norway; ^4^ Department of Oncology, Oslo University Hospital, Oslo, Norway; ^5^ Department of Radiation Oncology, University of California, Los Angeles, Los Angeles, CA, United States; ^6^ Department of Urology, University of California, Los Angeles, Los Angeles, CA, United States; ^7^ Institute of Clinical Medicine, University of Oslo, Oslo, Norway

**Keywords:** prostatic neoplasms, re-irradiation, image-guided radiotherapy, radiation dose hypofractionation, toxicity, treatment outcome

## Abstract

**Background:**

Up to half of patients with localized prostate cancer experience biochemical relapse within 10 years after definitive radiotherapy. The aim of this prospective study was to investigate the toxicity, dose to the organs at risk (OARs), and efficacy of dose-intensified focal salvage radiotherapy.

**Methods and Material:**

Thirty-three patients (median age 68.8 years) with histologically confirmed relapse after primary definitive radiotherapy were enrolled between 2012 and 2019. No patients had metastases at imaging or in bone marrow aspiration. Twenty-three patients were treated with high dose-rate brachytherapy to the recurrent tumor, defined at multiparametric MRI, with 3 fractions of 10 Gy with two weeks interval, and 10 patients by stereotactic body radiotherapy with 35 Gy to the local recurrence and 25 Gy to the whole prostate in 5 fractions. We used the RTOG-scoring system to grade genitourinary (GU) and gastrointestinal toxicity (GI) at three months (acute), and at 12, 24, and 36 months (late). Dose-volume histogram parameters to the local recurrence and the OARs were obtained and 2 Gy equivalent (EQD2) total dose was calculated using the linear-quadratic model with α/β = 3 Gy. Efficacy was assessed by the progression-free interval and overall survival.

**Results:**

Median follow-up time was 81 months (range 21–115). The cumulative moderate to severe GI and GU toxicities were 3.0% (1/33) and 15.2% (5/33). Six patients had grade 1 acute GI toxicity, none had grade 2 or 3. One patient had grade 3 acute GU toxicity, two had grade 2, and fourteen had grade 1. One patient had late GI toxicity grade 2 and eight had grade 1. Four patients had late GU toxicity grade 2 and eight had grade 1. No patients had grade 3 late toxicity. The mean total D90 to the recurrent tumor was 77.7 ± 17.0 Gy. The mean total rectum D2cc was 17.0 ± 7.9 Gy and the mean total urethra D0.1cc was 29.1 ± 8.2 Gy. Twenty-eight patients had re-irradiation without androgen deprivation therapy (ADT). Nine of these are still relapse-free and 10 had a recurrence-free interval longer than 2 years.

**Conclusion:**

The toxicity of salvage radiotherapy was mild to moderate. One-third of the patients achieved long-term stable disease without ADT and one-third had a recurrence-free interval longer than 2 years. Some patients progressed rapidly and probably did not benefit from re-irradiation.

## Introduction

The primary treatment options for localized prostate cancer are radiotherapy and radical prostatectomy. Biochemical relapse occurs in up to half of the patients (1–5). In contrast to the management of recurrence after prostatectomy, optimal management of recurrence after radiotherapy remains unclear due to the lack of large prospective studies in this setting (6). Even the management of a true local recurrence after definitive radiotherapy is controversial and consensus recommendation is limited (6).

Androgen deprivation therapy (ADT) is often used for radio-recurrence, but is non-curative and is associated with impaired quality of life. Local treatment could postpone the onset of ADT and thereby the development of castration-resistant disease, and potentially cure the patient (7).

Salvage prostatectomy has the longest history of use for local treatment of intra-prostatic recurrence but suffers from significant side effects. Re-irradiation has been considered to induce serious toxicity. However, more focal radiotherapies such as brachytherapy (BT) and stereotactic body radiotherapy (SBRT) are less invasive compared to prostatectomy and may circumvent the problem of overdosage to critical structures (6). This can be achieved by applying inhomogeneous dose patches that lower the dose to the whole gland and preferably re-irradiate the local recurrent tumor only. One major concern of applying salvage irradiation is that the tolerance dose to the urothelium and rectal mucosa may limit the sufficient dose delivered to the tumor. To avoid unacceptable toxicity one can use technical strategies that spare the urethra and the rectum.

A recent Delphi consensus paper investigated the expert opinion on salvage re-irradiation and reported increasing interest (8). A recent large meta-analysis reported that the genitourinary toxicity rate for re-irradiation, particularly for SBRT and high dose-rate (HDR)-BT, were significantly less than those reported after salvage prostatectomy, high-intensity focused ultrasound (HIFU), and cryotherapy (6, 9). To establish re-irradiation as a treatment option results from prospective studies with sufficient long follow-up is highly warranted (6, 7, 9–11).

Herein we report prospectively recorded acute and late gastrointestinal (GI) and genitourinary (GU) toxicity and long-term clinical outcome after re-irradiation with HDR-BT and SBRT.

## Materials and Methods

### Study Population

The Regional Committee for Medical Research Ethics southeast Norway approved this prospective study of focal salvage re-irradiation (2011/954) and all patients provided written informed consent.

The main inclusion criteria were local recurrence after primary curative intended external beam radiotherapy (EBRT), defined as prostate-specific antigen (PSA) >nadir + 2 ng/ml, and no metastases neither at imaging nor in bone marrow aspiration samples. Further inclusion criteria were PSA <10 ng/ml, PSA doubling time >6 months, more than 2 years recurrence-free interval since primary radiotherapy, and ECOG 0–1 with a life expectancy >5 years.

Between 2012 and 2019, we included 33 patients previously treated with conformal RT of 70–78 Gy to the prostate and seminal vesicles (**Table 1**). At primary treatment, the patients were diagnosed with intermediate (n = 13) or high-risk disease (n = 20) according to the D’Amico risk classification system (12). At recurrence, all patients had multiparametric MRI of the pelvis and lower lumbar spine, 24 had FACBC PET/CT (trans-1-amino-3-^18^F-fluorocyclobutanecarboxylic-acid positron emission tomography/computed tomography) and two had PSMA PET/CT (prostate-specific membrane antigen) to localize the recurrence and exclude metastatic disease. Intra-prostatic tumor recurrence was histologically verified in all but one patient.

**Table 1:** Detailed overview of patient characteristics and treatment.

ID	Primary diagnosis				Primary treatment		Primary RT to PSA recurrence (months)	Recurrence to re-irradiation (months)	At salvage re-irradiation				Salvage re-irradiation	
	GS	T	iPSA (ng/ml)	D´Amico risk classification^*^	RT	ADT			Age (years)	PSA (ng/ml)	IPSS	Comorbidity	RT and dose (Gy)	ADT
1	4 + 3	T2	14.7	Intermediate	74	No	50	23	71	6.4	12	Hypertension	HDR-BT 3 × 10 Gy	No
2	3 + 4	T2	8.0	Intermediate	74	No	52	9	66	4.2	4	Other cancer	HDR-BT 3 × 10 Gy	No
3	3 + 4	T2	10	Intermediate	74	>1 yr.	77	6	68	2.8	4	None	HDR-BT 3 × 10 Gy	No
4	3 + 4	T1c	22	High	74	3 months	43	17	65	3.0	20	Arrhythmia	HDR-BT 3 × 10 Gy	No
5	4 + 5	T3b	59	High	74	>1 yr.	60	9	58	2.3	12	Diabetes	HDR-BT 3 × 10 Gy	No
6	3 + 3	T1c	42	High	74	>1 yr.	101	6	66	4.0	6	None	HDR-BT 3 × 10 Gy	No
7	4 + 3	T3b	66	High	74	>1 yr.	42	4	68	3.8	3	Cerebral insult	HDR-BT 3 × 10 Gy	No
8	2 + 3	T1c	11.3	Intermediate	74	No	152	3	67	5.5	3	Hypertension	HDR-BT 3 × 10 Gy	No
9	4 + 5	T3b	58	High	74	>1 yr.	29	1	66	3.3	0	Other cancer	HDR-BT 3 × 10 Gy	No
10	3 + 3	T2	18	Intermediate	74	No	126	4	65	4.5	NA	None	HDR-BT 3 × 10 Gy	No
11	5 + 4	T3b	39	High	74	>1 yr.	35	4	70	7.9	6	None	HDR-BT 3 × 10 Gy	Short
12	4 + 3	T3a	45	High	74	>1 yr.	73	8	68	4.5	4	None	HDR-BT 3 × 10 Gy	No
13	3 + 4	T2	13	Intermediate	74	No	114	10	64	6.5	NA	Hypertension	HDR-BT 3 × 10 Gy	No
14	3 + 3	T1c	12	Intermediate	74	6 months	143	9	70	7.2	1	Diabetes	HDR-BT 3 × 10 Gy	No
15	3 + 4	T2a	4.5	Intermediate	74	6 months	63 ^3^	4	68	1.6	5	Hypertension	HDR-BT 3 × 10 Gy	No
16	3 + 4	T3a	28	High	74	>1 yr.	95	15	63	8.5	2	Hypertension	HDR-BT 3 × 10 Gy	No
17	3 + 4	T2	30	High	70	No	77	18	72	9.6	5	Arrhythmia	HDR-BT 3 × 10 Gy	No
18	4 + 4	T3b	17	High	74	>1 yr.	64	3	75	6.4	7	Diabetes	HDR-BT 3 × 10 Gy	No
19	3 + 4	T2	20	Intermediate	70	>1 yr.	41	7	70	4.2	1	Hypertension	HDR-BT 3 × 10 Gy	No
20	3 + 3	T1c	10	Intermediate	74	No	76	7	73	6.5	13	Hypertension	HDR-BT 3 × 10 Gy	No
21	3 + 4	T3a	5.4	High	74	>1 yr.	113	12	72	4.7	5	Arrhythmia	HDR-BT 3 × 10 Gy	No
22	3 + 3	T2	15.5	Intermediate	74	No	122	4	74	4.0	9	Hypertension	HDR-BT 3 × 10 Gy	3 months
23	4 + 4	T3a	9.4	High	74	>1 yr.	62	6	77	4.1	2	Hypertension	HDR-BT 3 × 10 Gy	3 months
24	4 + 4	T3a	17	High	74	>1 yr.	72	6	69	5.0	2	None	SBRT 7(5) Gy × 5	No
25	3 + 4	T2	8.0	Intermediate	78	6 months	84	8	73	3.5	20	Diabetes	SBRT 7(5) Gy × 5	No
26	3 + 5	T3a	70	High	74	>1 yr.	122	2	74	7.8	2	None	SBRT 7(5) Gy × 5	No
27	3 + 4	T3a	6.4	High	70	6 months	111	4	74	1.9	NA	Hypertension	SBRT 7(5) Gy × 5	No
28	3 + 4	T2	44	High	74	>1 yr.	65 ^3^	7	78	2.5	2	Hypertension	SBRT 7(5) Gy × 5	No
29	4 + 4	T3b	7.6	High	74	>1 yr.	80	5	67	0.83 ^1^	5	Other cancer	SBRT 5 Gy × 6	>1 yr.
30	4 + 3	T3b	29	High	74	>1 yr.	85	4	75	0.31 ^2^	3	None	SBRT 7(5) Gy × 5	6 months
31	4 + 3	T3a	10	High	74	>1 yr.	55	3	67	2.8	11	Arrhythmia	SBRT 7(5) Gy × 5	No
32	4 + 3	T2	8	Intermediate	74	>1 yr.	61	6	72	3.9	2	Diabetes	SBRT 5 Gy × 5 ^4^	No
33	4 + 4	T3b	37	High	74	>1 yr.	52	15	76	4.6	12	None	SBRT 7(5) Gy × 5	No

^*^D’Amico et al. (12).

^1^Had five months of ADT before SBRT.

^2^Had two months of ADT before SBRT.

^3^Recurrence based on MRI and biopsy, not PSA. Date of recurrence is the biopsy date.

^4^Received 5 fractions without a boost to the recurrent tumor.

GS, Gleason score; T, T-stage; PSA, Prostate specific antigen; iPSA, initial PSA; ADT, androgen deprivation treatment; IPSS, The International Prostate Symptom Score; RT, radiotherapy; SBRT, stereotactic body radiotherapy; HDR-BT, high dose-rate brachytherapy; NA, not applicable.

**Figure 1:**
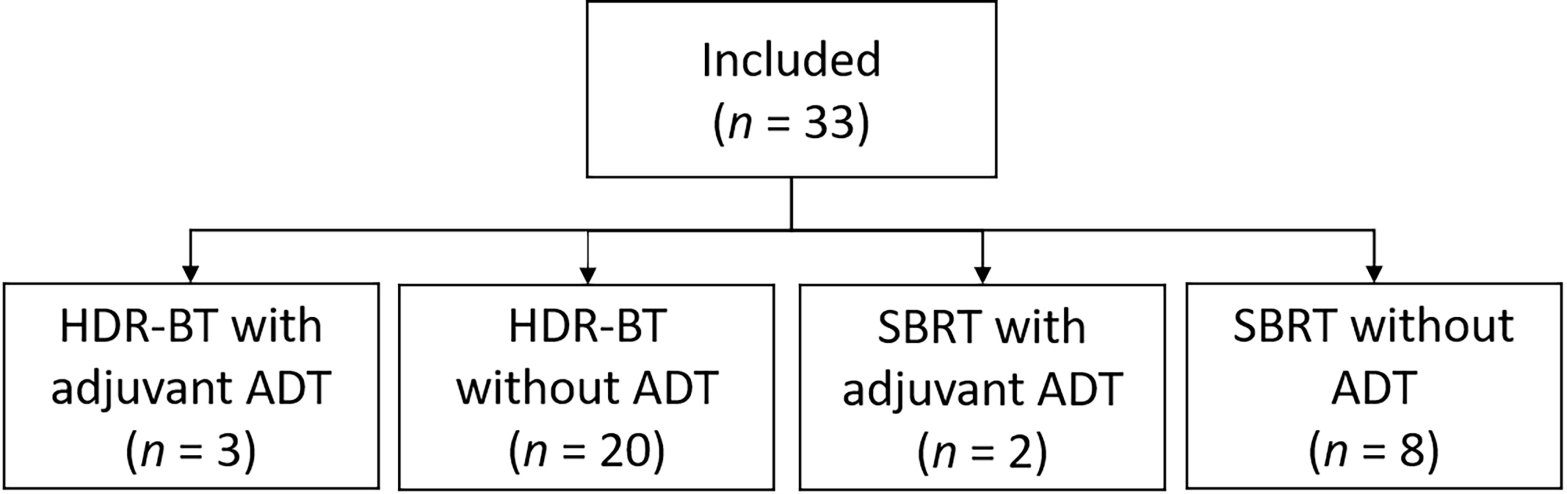
Overview of the salvage treatment. HDR-BT, high dose-rate brachytherapy; ADT, androgen deprivation therapy; SBRT, stereotactic body radiotherapy.

The median age of the study population was 69.8 years (interquartile range (IQR) 6.8), and the median PSA was 4.1 ng/ml (IQR 3.8). The median time from primary radiotherapy to biochemical recurrence was 73.0 months (IQR 52.5). Twenty-eight patients were eugonadal at salvage re-irradiation while five patients received either ongoing or concomitant ADT (**Figure 1**). The first 23 patients received HDR-BT, and the last 10 patients received SBRT. A detailed overview of the study population and treatment is shown in **Table 1**.

### Imaging

Multiparametric MRI prior to salvage re-irradiation included morphological T2-weighted, diffusion-weighted (DW), and dynamic contrast enhanced (DCE) sequences of the prostate. The acquisition protocol is described in detail in Tulipan et al. (13). The technical standard of the imaging protocol was in accordance with current technical requirements for prostate MRI (14). One radiologist with more than nine years of experience in prostate cancer MRI (KH) prospectively interpreted the examinations for study inclusion and biopsy guidance. The other radiologist (UR) with four years of experience retrospectively reviewed all the MRI examinations both from primary diagnosis, if available, and from the time of recurrence, with the purpose of comparing the site of the primary and recurrent tumor.

### Planning and Treatment Techniques

**Figure 2:**
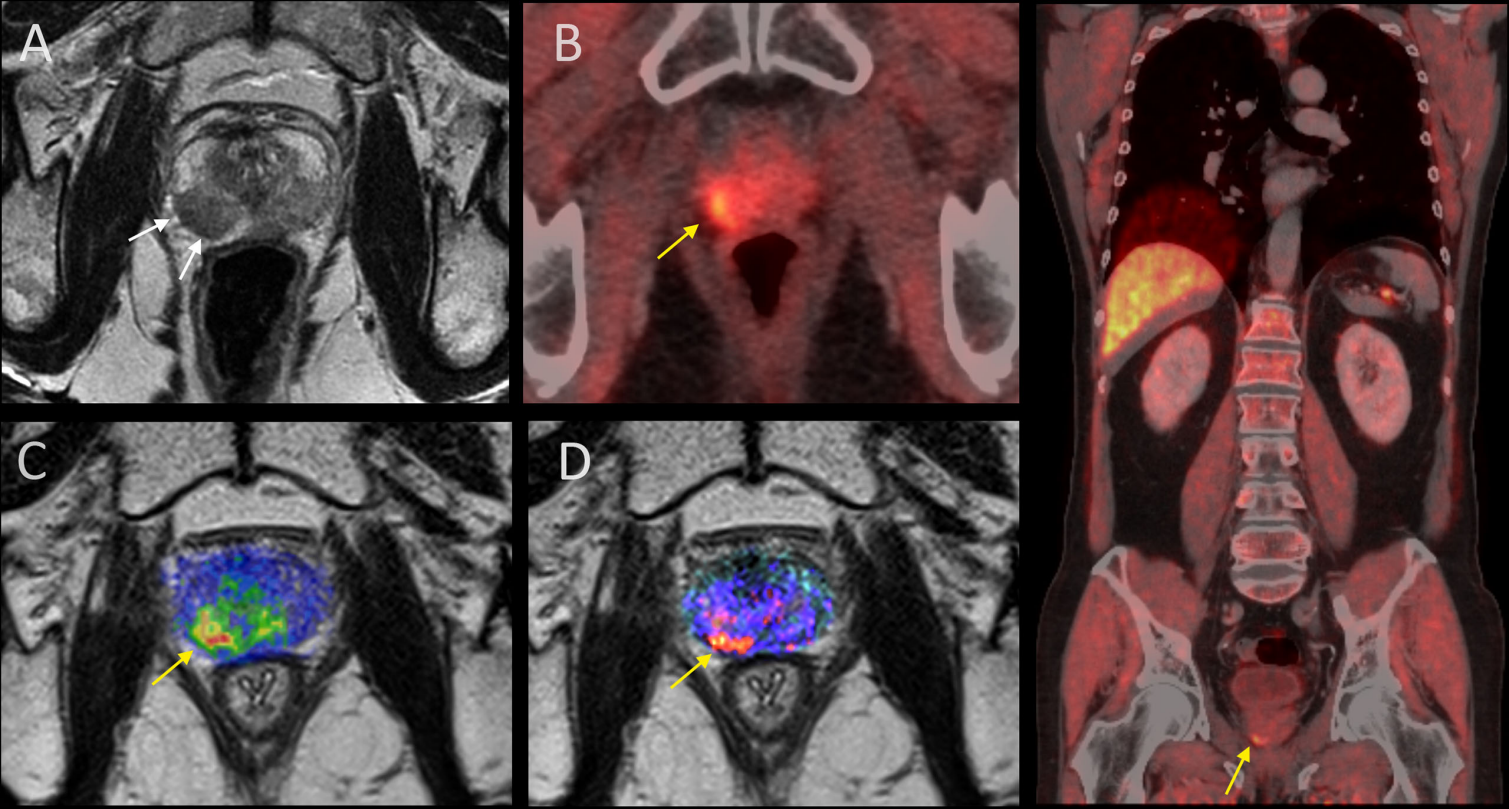
Primary and radio-recurrent prostate cancer at imaging. **(A)** Primary tumor at T2W MRI (white arrows). **(B–D)** Recurrent tumor (yellow arrow) at FACBC PET/CT, diffusion weighting overlaid on T2W MRI, and dynamic contrast-enhanced MRI overlaid on T2W MRI. To the right: Whole-body FACBC PET/CT to prove true local recurrence only (yellow arrow).

**Figure 3:**
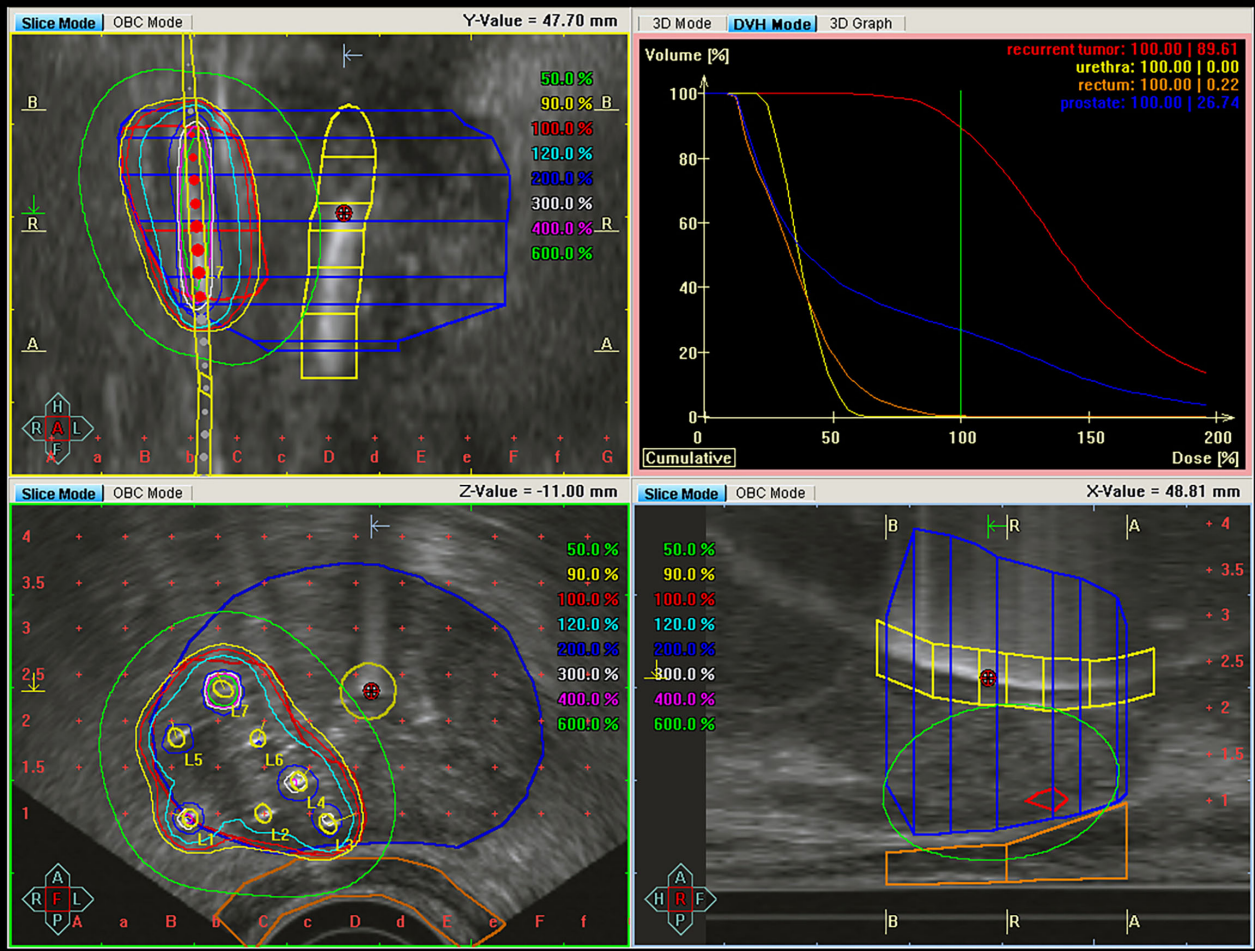
Dose distribution for high dose-rate brachytherapy of the same patient as in **Figure 2** showing the coronal (upper left), transversal (lower left), and sagittal (lower right) plane. The recurrent tumor (red), prostate (blue), urethra (yellow), and rectum wall (brown) are delineated. Air-filled gel has been inserted in the urethra catheter to visualize the urethra in the ultrasound images. The dose-volume histograms (upper right quadrant) show the highly conformal dose distribution, sparing the rectum and urethra, achieved by brachytherapy.

Twenty-three patients received mainly focal HDR-BT in three fractions every second week using the microSelectron HDR 192Ir source (Nucletron B.V., Veenendaal, The Netherlands). The planning aim was 10 Gy to the gross tumor volume (GTV), which for the majority of the patients (n = 20) was defined as the recurrent tumor identified at imaging (**Figure 2**). The HDR-BT procedure has previously been described in detail in Raabe et al. (15). In short, the patients were under general anesthesia and in the lithotomy position. A Foley catheter was placed in the bladder. Guided by transrectal ultrasound (US) the needles were inserted into the gross tumor volume (GTV) through the perineum. The recurrent tumor, prostate gland, rectal wall, and the urethra (cylinder with a radius of 3 mm) were delineated by the oncologist on US images acquired both before and after needle insertion (**Figure 3**). The delineation of the recurrent tumor was guided by multiparametric MRI (**Figure 2**). Intra-operative treatment planning, namely, inverse plan optimization and consecutive graphical adjustments, was performed using Oncentra Prostate Vs.4.1.6 (Nucletron). Source positions 3 mm or closer to the urethra were not allowed to be used. Initial optimization settings and dose constraints are found in **Supplementary Tables 1–3**.

For patients 9, 11, and 23 (**Table 1**), the whole prostate gland, excluding the urethra, was defined as the GTV. For patient 23, the GTV included only the recurrent tumor on the two last fractions.

Ten patients received SBRT with 6MV flattening filter-free volumetric arc therapy (VMAT) delivered on a linear accelerator (Varian TrueBeam ™ STx, Varian Medical Systems, Palo Alto, USA). Fiducial gold markers implanted as strands along the urethra and as cubes in the prostate prior to treatment planning assisted the image-guided RT (daily cone-beam CT for target positioning and verification). The SBRT was delivered in five fractions as a simultaneous integrated boost with a planning aim of 35 Gy to the recurrent tumor and 25 Gy to the prostate gland (n = 8) or in six fractions with a planning aim of 30 Gy to the whole prostate gland (n = 1). One patient (patient 32) received only five fractions without an integrated boost to the tumor. The fractions were delivered every other day. The delineation of target volumes (recurrent tumor and/or prostate gland) and organs at risk (OARs) (urethra, bladder, rectum, anal canal, and femoral heads), and treatment planning was performed using Raystation 5 (RaySearch Laboratories, Stockholm, Sweden). An isotropic planning target volume (PTV) margin of 3 mm was used for both the recurrent tumor and the prostate. The treatment plans were optimized with a steep dose gradient to the urethra and normalized to a prescription volume that excluded the urethra with a margin of 3–5 mm. Clinical goals and dose constraints are found in **Supplementary Tables 4, 5**. Patient-specific quality assurance of the SBRT plans was performed prior to the onset of treatment using the ArcCheck^®^ phantom (Sun Nuclear Corporation, Melbourne, USA).

Dose to 90% (D90) of the target volumes (recurrent tumor/prostate) were found from the dose volume histograms. Also, the minimum dose to the most exposed 2 and 0.1 cubic centimeter (D2cc and D0.1cc) were recorded for the rectum and urethra, respectively. The linear-quadratic model was used to calculate 2 Gy equivalent (EQD2) total dose, assuming α/β = 3 Gy for both the recurrent tumor/prostate and for the OARs.

### Toxicity and Clinical Outcome

We used the toxicity criteria of the Radiation Therapy Oncology Group (RTOG) (16) to grade gastrointestinal (GI) and genitourinary (GU) toxicity at three months (acute), and at 12, 24, and 36 months (late). RTOG-grading ceased if patients received additional treatment such as HIFU, prostatectomy, or chemotherapy.

Patients were followed every three months for the first two years, and every six months for the following years. The clinical outcome was measured as the recurrence-free interval from re-irradiation to the second recurrence, defined as PSA >2 ng/ml above nadir after salvage re-irradiation (8).

### Data Analysis

Data are presented with descriptive statistics. We calculated Kaplan–Meier estimates for recurrence-free and overall survival after re-irradiation. To assess the association between dose and late toxicity, we selected the highest RTOG-scoring of the three reported late time points. We performed subgroup analyses to investigate parameters that could identify the patients who had the highest benefit from salvage re-irradiations. Data were analyzed and figures created using Prism 6 for Mac OS X version 6.0f (GraphPad Software Inc., San Diego, CA).

## Results

### Toxicity

**Figure 4:**
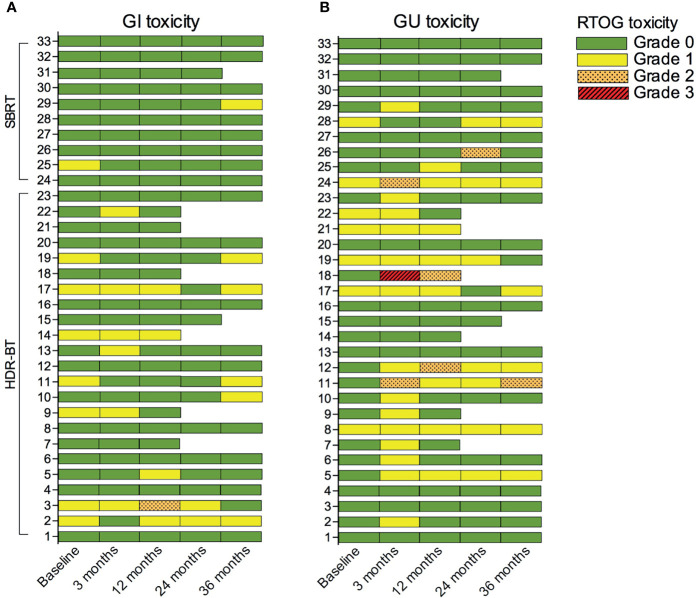
RTOG-graded gastrointestinal (GI) **(A)** and genitourinary (GU) **(B)** toxicity before (baseline) and following salvage re-irradiation. RTOG grading ceased if patients received additional local treatment such as HIFU, prostatectomy, or chemotherapy. HDR-BT, High dose-rate brachytherapy; SBRT, Stereotactic body radiotherapy.

**Figure 4** shows the course of RTOG-graded toxicity for each patient prior to, and 3, 12, 24, and 36 months after salvage re-irradiation. The cumulative moderate to severe GI and GU toxicities were 3.0% (1/33) and 15.2% (5/33). Before re-irradiation, 8 had grade 1 GI toxicity, and 7 had grade 1 GU toxicity. At 3 months, six patients had grade 1 acute GI toxicity while none had grade 2 or 3. Fourteen patients had grade 1 acute GU toxicity, two had grade 2, and one had grade 3. At later time points, eight patients had late GI toxicity grade 1, and one had grade 2. Eight had grade 1 late GU toxicity and four had grade 2. No patients had grade 3 late GI or GU toxicity.

Eight patients received additional treatment after re-irradiation. The remaining 25 patients had toxicity scored at 36 months. Compared to baseline, only two (patients 10 and 29) reported increased GI toxicity, and three (patients 5, 11, and 12) reported increased GU toxicity. Patient 18, who experienced severe toxicity, had poorly regulated diabetes.

**Figure 5:**
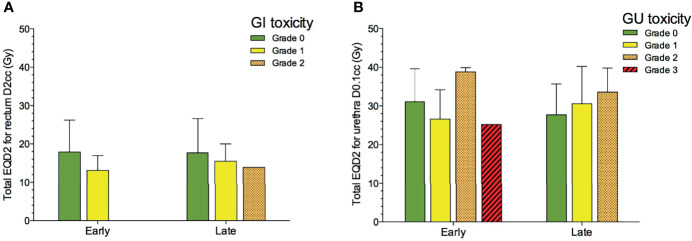
Total EQD2 for rectum D2cc **(A)** and urethra D0.1cc **(B)** from the re-irradiation and RTOG-graded gastrointestinal (GI) and genitourinary (GU) toxicity after salvage re-irradiation (n = 33). Bars represent mean values, and whiskers SD.

The mean total D90 to the local recurrence was 77.7 ± 17.0 Gy. The mean total D2cc for the rectum was 17.0 Gy (SD 7.9), and the mean total D0.1cc for the urethra was 29.1 Gy (SD 8.2). Only the contribution from the re-irradiation is included in these figures. We found no association between doses to rectum and urethra from the re-irradiation, and toxicity (**Figure 5**).

### Clinical Outcome

**Figure 6:**
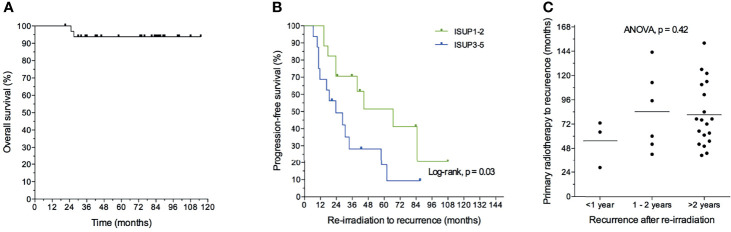
Kaplan–Meier estimate of overall survival **(A)** and progression-free survival **(B)** after re-irradiation without androgen deprivation treatment (n = 28). Time to first recurrence for patients recurring within one, two, or more than two years after re-irradiation **(C)**.

**Figure 7:**
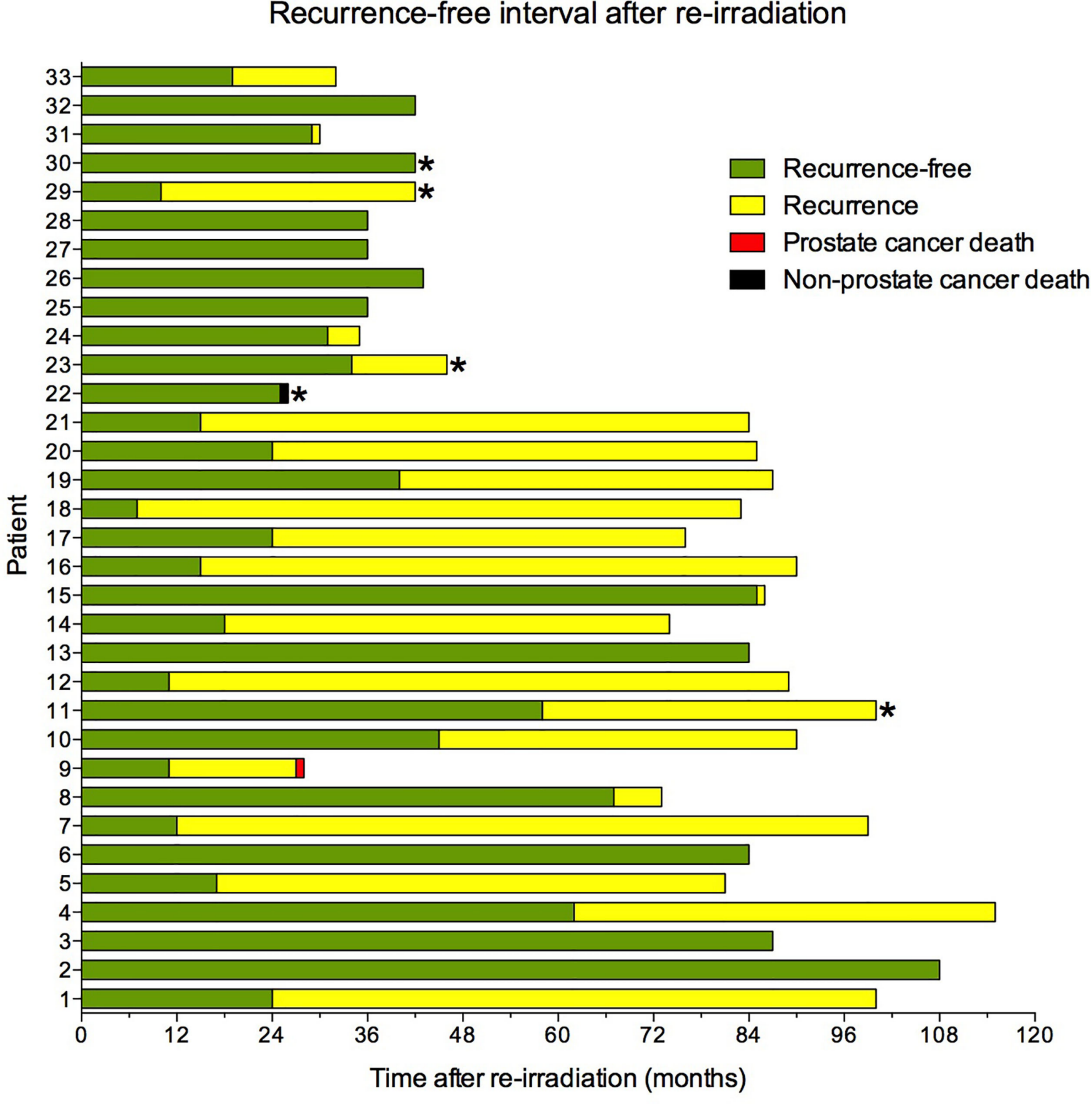
Recurrence-free interval after re-irradiation. *Patients who received androgen deprivation treatment at salvage re-irradiation.

During the median follow-up after re-irradiation of 81 months (range 21–115), two patients died, one of prostate cancer and one of complications following aortic dissection (**Figure 6A**). For the entire cohort, the median biochemical progression-free survival after re-irradiation was 40 months. For the 22 patients who relapsed, the median time to secondary recurrence was 24 months (range 7–85). Data for individual patients are reported in **Figure 7**. Eleven patients, including the patient who died of complications, did not relapse, whereas 22 had a second recurrence, four within one year and six within the second year. Twelve patients had a recurrence-free interval longer than two years.

Twenty-eight patients (85%) had salvage radiotherapy without ADT. Nine of these 28 (32%) are still relapse-free, 10 (36%) had a recurrence-free interval longer than two years, and nine (32%) patients relapsed within the two years.

The clinical goal of re-irradiation is to eradicate the recurrent tumor and/or to postpone the onset of ADT. Some patients had a short recurrence-free interval after re-irradiation and probably limited clinical benefit. We sought to identify predictive parameters and hypothesized that less aggressive tumors would benefit the most. We therefore investigated whether the short time from primary radiotherapy to recurrence and low ISUP (International Society of Urological Pathology) grade groups, were markers of a long-term effect of salvage re-irradiation. No other clinical parameters were significant.

The median PFS after re-irradiation was 67 months for patients with ISUP grade groups 1–2 compared to 24 months for ISUP grade groups 3–5. Patients with ISUP grade groups 1–2 had significantly longer time from re-irradiation to recurrence and thus longer progression-free survival (**Figure 6B**, log-rank test; p = 0.03). However, time from primary radiotherapy to recurrence was not a significant marker of early recurrence after re-irradiation (**Figure 6C**).

### Site of Recurrence

Sixteen patients had MRI both at primary diagnosis and at first recurrence. For 15 of these patients, the recurrent tumor occurred within the extent of the primary tumor (**Figure 2**). The extent of the primary tumor for the last patient could not be assessed due to artifacts from air in the rectum.

## Discussion

This prospective study reports toxicity and long-term clinical outcome after salvage re-irradiation of localized intra-prostatic recurrence. The study cohort consisted of 33 patients who initially were treated with primary EBRT (70–78 Gy). All had a true local recurrence verified by biopsy and no metastases at imaging or in bone marrow samples. For most patients (28/33) salvage re-irradiation was delivered without androgen deprivation therapy. Overall, the GU and GI toxicity was mild to moderate. One-third of the patients had a biochemical relapse within the first two years, one third relapsed later than two years, and one-third of the patients are still relapse-free.

There are several studies that have reported results from salvage re-irradiation (6, 7, 10, 11, 17). However, there is a large heterogeneity among the reported studies: different primary treatment (prostatectomy, cryotherapy, HIFU, low dose-rate (LDR) brachytherapy, EBRT, and combinations), limited follow-up time, peri-salvage use and inconsistent reporting of ADT, and retrospective study design. The limited GU/GI toxicity in our patients is in line with the reasonable toxicity reported in the prospective phase II RTOG-0526 trial (17) and two recent large systematic reviews and meta-analyses (6, 7).

In the RTOG-0526 applying salvage LDR-BT 14% had grade 3 GU toxicity compared to 3% in our study. Because only one patient in our study had grade 3 GU toxicity, the data is too sparse to assess if this was associated with primary and salvage treatments. The patients in the RTOG-0526 study received 78 Gy/39 fractions or 81 Gy/45 fractions, a slightly higher dose than in our cohort. Nearly all patients in the RTOG-0526 study had whole-gland salvage LDR brachytherapy, whereas we used focal HDR-BT/SBRT and delivered a boosted dose to the recurrent tumor with pre-specified low tolerance dose to the urethra (**Supplementary Tables 3**, **5**).

The 2-year biochemical recurrence-free survival was 68% (19 of 28 patients). In the review from Valle et al. 2-year recurrence-free survival was 62% for SBRT and 77% for HDR-BT, however, about 40% of these patients received peri-salvage ADT (6). Corkum et al. (7) reported the random effect of biochemical recurrence-free survival (BRFS) to be 60% with a significant heterogeneity (50–70%). Crook et al. have recently published their long-term clinical outcome of the RTOG-0526 trial and found a 5-year disease-free survival of 61%. Their results seem in line with ours, but they had longer follow-up time, excluded high-risk patients, and permitted up to six months of peri-salvage ADT. The majority of our patients had high-risk disease.

In the setting of radio-recurrence, salvage prostatectomy and ADT are the guideline-recommended standard options. A longstanding principle in radiation oncology is that after EBRT, re-irradiation will exceed normal tissue tolerances leading to potentially serious toxicity (7–9). A recent ESTRO ACROP consensus paper agrees that re-irradiation is a feasible therapeutic option for selected patients (8). The meta-analyses from Valle et al. reported significantly less GU toxicity rates for SBRT and BT than after prostatectomy, HIFU, and cryotherapy (6, 9). In the current study, we demonstrate that re-irradiation, without rectal spacer devices, is feasible and tolerable provided stringent dose constraints to the urethra and rectum (**Supplementary Tables 3**, **5**). In the future, the assessment of germline variants that predict clinical radio-sensitivity could be implemented to improve patient selection and further reduce toxicity (18).

The therapeutic goal of re-irradiation is the eradication of the recurrence or substantial delay of onset of ADT and subsequent development of castration-resistant disease. One-third of the patients in our study had an early second biochemical relapse within two years, indicating that not all patients will benefit from re-irradiation. The only marker of poor clinical outcome was the high ISUP grade group of the primary tumor. Two-thirds of the patients saw clinical benefits, more than 2-years BFS, probably because the patients were carefully selected by strict inclusion criteria and thorough imaging to localize the site and extent of the recurrence and exclude metastatic disease. The ESTRO APCO Delphi consensus also agrees on and highlights the need for strict inclusion criteria and state-of-the-art imaging (8).

All recurrences occurred within the extent of the primary tumor for the 15 patients in which we had MRI with sufficient image quality at primary diagnosis. Jalloh et al. (19) also found that nearly all recurrences were within the extent of the primary index tumor. These findings indicate that the radiotherapy of primary prostate cancer could be improved by dose escalation (20). Modern dose painting techniques may deliver increased dose selectively to the radio-persisting intra-prostatic lesion without increasing the dose to the OARs (21).

The major limitations of our study are the limited sample size and the lack of a control group. The minor limitations are that not all patients had the same treatment, some had HDR-BT, some had SBRT, and five patients received peri-salvage ADT. The major strengths are the prospective design, clearly defined inclusion criteria, modern ultra-hypofractionated image-guided radiotherapy, a long follow-up time, and state-of-the-art imaging. Furthermore, most of the patients (28/33) did not have ADT. As such, our prospective study does not provide a high level of evidence but adds to the body of knowledge.

## Conclusion

Re-irradiation of intra-prostatic recurrence with HDR-BT and SBRT is feasible and resulted in mild to moderate GU or GI toxicity. Two-thirds of the patients experienced more than 2-years BFS, one-third are still recurrence-free without ADT. Some patients progressed rapidly and may not have benefitted from salvage radiotherapy. Careful selection of patients is needed.

## Data Availability Statement

The original contributions presented in the study are included in the article/**Supplementary Material**. Further inquiries can be directed to the corresponding author.

## Ethics Statement

The studies involving human participants were reviewed and approved by The Regional Committee for Medical Research Ethics South East Norway. The patients/participants provided their written informed consent to participate in this study.

## Authors Contributions

Conceptualization, TS, KH, AK, and WL. Methodology, UR, TS, LN, TH, LD, HG, KH, and WL. Formal analysis, TS. Investigation, UR, TS, LN, TH, LD, HG, JH, KH, and WL. Resources, WL. Writing—Original Draft, UR, TS, KH, and WL. Writing—Review & Editing, UR, TS, LN, TH, LD, HG, JH, AK, KH, and WL. Visualization, TS and KH. Supervision, TS, KH, and WL. Funding Acquisition, WL. All authors listed have made a substantial, direct, and intellectual contribution to the work and approved it for publication.

## Funding

We would like to thank the Raagholt Foundation for their generous support.

## Conflict of Interest

The authors declare that the research was conducted in the absence of any commercial or financial relationships that could be construed as a potential conflict of interest.

## Publisher’s Note

All claims expressed in this article are solely those of the authors and do not necessarily represent those of their affiliated organizations, or those of the publisher, the editors and the reviewers. Any product that may be evaluated in this article, or claim that may be made by its manufacturer, is not guaranteed or endorsed by the publisher.
