# Supplementary material for: A Prospective Study of High Dose-Rate Brachytherapy or Stereotactic Body Radiotherapy of Intra-Prostatic Recurrence: Toxicity and Long Term Clinical Outcome
Source: Front Oncol. 2022 Apr 5;12:861127. doi: 10.3389/fonc.2022.861127 (PMC9022104; doi:10.3389/fonc.2022.861127)
Supplement: Supplementary file 1 [file Table_1.docx]

Supplementary Material

**1. HIGH DOSE-RATE BRACHYTHERAPY (HDR-BT)**

**Supplementary Table 1**: Delineation of organs

| Organ | Description | Type |
| --- | --- | --- |
| Prostate | The whole prostate gland | CTV |
| Recurrent tumor | The recurrent tumor volume defined on MRI | GTV |
| Rectum | The anterior rectal wall in the full length of the prostate | OAR |
| Urethra | Cylinder with radius 3mm in the full length of the prostate (Foley-catheter) | OAR |

CTV = Clinical target volume, GTV = Gross tumor volume, OAR = Organ at risk

**Supplementary Table 2**: Initial settings for inverse dose volume histogram-based optimization (DVHO)

| Organ | Dose limit (%) | Impact factor |
| --- | --- | --- |
| Normal tissue | 130 | 0.1 |
| GTV | Minimum 100  Maximum 150 | 0.5  0.1 |
| Rectum | 50 | 0.1 |
| Urethra | 50 | 0.5 |

Graphical optimization was performed after inverse DVHO

GTV = Gross tumor volume

**Supplementary Table 3**: Clinical goals

| Organ | Dose constraint/objective |
| --- | --- |
| GTV | V100 ≥ 90% (mandatory), V100 ≥ 95% (optimal), D90 ≥ 100% (mandatory) |
| Rectum | D2cc: Maximum 4 Gy (as low as possible) |
| Urethra | D0.1cc: Maximum 5 Gy (as low as possible) |

GTV = Gross tumor volume

**2. STEREOTACTIC BODY RADIOTHERAPY (SBRT)**

**Supplementary Table 4**: Delineation of organs

| Organ | Description | Type |
| --- | --- | --- |
| Prostate | The whole prostate gland. | GTV |
| Reccurrent tumor | The recurrent tumor volume defined on MRI | GTV_T |
| Anal canal | The anal canal |  |
| Rectum | From the dentate line to the rectosigmoid junction | OAR |
| Bladder | The whole bladder, including the bladder wall | OAR |
| Urethra^*^ | Delineated based on MRI and the fiducial markers, extending at least 5 mm below the prostate and 2 mm into the bladder | OAR |
| Femoral heads | Both femoral heads | OAR |

^*^An isotropic 3 mm PRV (Planning organ at risk volume) to the urethra was used.

CTV = Clinical target volume, GTV = Gross tumor volume, OAR = Organ at risk.

**Supplementary Table 5**: Clinical goals

| Organ | Dose constraint/objective |
| --- | --- |
| GTV | V100 ≥ 90% (mandatory),V100 ≥ 95% (optimal), D90 ≥ 100% (mandatory) |
| PTV | V100 ≥ 90% (mandatory), V100 ≥ 95% (optimal), D90 ≥ 100% (mandatory) |
| Rectum | D0.5cc < 25 Gy, D30% < 14 Gy |
| Urethra | D0.1cc < 25 Gy |
| PRV_Urethra | D0.1cc < 30 Gy, D0.05cc < 25 Gy |
| Bladder | D2cc < 25 Gy (mandatory), D0.05cc < 25 Gy (optimal), D15cc < 18.3 Gy |

GTV = Gross target volume, PTV = Planning target volume, PRV = Planning organ at risk volume

**
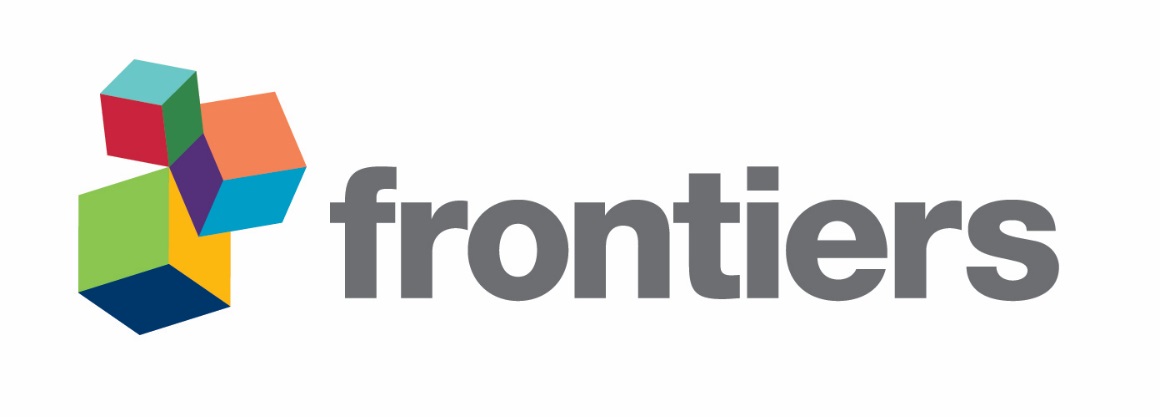
**
